# Frequency and spectrum of mitochondrial 12S rRNA variants in 440 Han Chinese hearing impaired pediatric subjects from two otology clinics

**DOI:** 10.1186/1479-5876-9-4

**Published:** 2011-01-04

**Authors:** Zhisen Shen, Jing Zheng, Bobei Chen, Guanghua Peng, Ting Zhang, Shasha Gong, Yi Zhu, Chuqin Zhang, Ronghua Li, Li Yang, Jianjin Zhou, Ting Cai, Lihua Jin, Jianxin Lu, Min-Xin Guan

**Affiliations:** 1 Department of Otolaryngology, Ningbo Medical Center, Li Huili Hospital, Ningbo, Zhejiang, China; 2 Attardi Institute of Mitochondrial Biomedicine and Zhejiang Provincial Key Laboratory of Medical Genetics, School of Life Sciences, Wenzhou Medical College, Wenzhou, Zhejiang, China; 3 Department of Otolaryngology, the Second Affiliated Hospital, Wenzhou Medical College, Wenzhou, Zhejiang, China; 4 Department of Otolaryngology, Yuyao People's Hospital, Yuyao, Zhejiang, China; 5 Department of Otolaryngology, the First Affiliated Hospital, Wenzhou Medical College, Wenzhou, Zhejiang, China; 6 Department of Human Genetics, Cincinnati Children's Hospital Medical Center, Cincinnati, Ohio 45229, USA; 7 Deparment of Pediatrics, University of Cincinnati College of Medicine, Cincinnati, Ohio, USA

## Abstract

**Background:**

Aminoglycoside ototoxicity is one of the common health problems. Mitochondrial 12S rRNA mutations are one of the important causes of aminoglycoside ototoxicity. However, the incidences of 12S rRNA mutations associated with aminoglycoside ototoxicity are less known.

**Methods:**

A total of 440 Chinese pediatric hearing-impaired subjects were recruited from two otology clinics in the Ningbo and Wenzhou cities of Zhejiang Province, China. These subjects underwent clinical, genetic evaluation and molecular analysis of mitochondrial 12S rRNA. Resultant mtDNA variants were evaluated by structural and phylogenetic analysis.

**Results:**

The study samples consisted of 227 males and 213 females. The age of all participants ranged from 1 years old to 18 years, with the median age of 9 years. Ninety-eight subjects (58 males and 40 females) had a history of exposure to aminoglycosides, accounting for 22.3% cases of hearing loss in this cohort. Molecular analysis of 12S rRNA gene identified 41 (39 known and 2 novel) variants. The incidences of the known deafness-associated 1555A > G, 1494C > T and 1095T > C mutations were 7.5%, 0.45% and 0.91% in this entire hearing-impaired subjects, respectively, and 21.4%, 2% and 2% among 98 subjects with aminoglycoside ototoxicity, respectively. The structural and phylogenetic evaluations showed that a novel 747A > G variant and known 839A > G, 1027A > G, 1310C > T and 1413T > C variants conferred increased sensitivity to aminoglycosides or nonsyndromic deafness as they were absent in 449 Chinese controls and localized at highly conserved nucleotides of this rRNA. However, other variants were polymorphisms. Of 44 subjects carrying one of definite or putative deafness-related 12S rRNA variants, only one subject carrying the 1413T > C variant harbored the 235DelC/299DelAT mutations in the *GJB2 *gene, while none of mutations in *GJB2 *gene was detected in other 43 subjects.

**Conclusions:**

Mutations in mitochondrial 12S rRNA accounted for ~30% cases of aminoglycoside-induced deafness in this cohort. Our data strongly support the idea that the mitochondrial 12S rRNA is the hot spot for mutations associated with aminoglycoside ototoxicity. These data have been providing valuable information and technology to predict which individuals are at risk for ototoxicity, to improve the safety of aminoglycoside antibiotic therapy, and eventually to decrease the incidence of deafness.

## Background

Aminoglycosides, such as gentamicin and tobramycin, are of great clinical importance for the treatment of bacterial infections. The use of these drugs can frequently lead to toxicity, which involves the renal, auditory and vestibular systems [1,2]. The renal impairment is usually reversible, whereas the auditory and vestibular ototoxicity is usually irreversible. In familial cases of ototoxicity, aminoglycoside hypersensitivity is often maternally transmitted, suggesting that mutation(s) in mitochondrial DNA (mtDNA) is one of molecular bases for this susceptibility [1,2]. As mitochondrial ribosomes share more similarities to bacterial ribosomes than do cytosolic counterparts, the human mitochondrial 12 rRNA was proposed to be the primary targeting site for aminoglycosides [3,4]. The mutational analysis of mitochondrial genome in several Chinese and Arab-Israeli families with maternally transmitted aminoglycoside ototoxicity or/and nonsyndromic deafness led to the landmark discovery of the 12S rRNA 1555A > G mutation in 1993 [3]. Subsequently, the 1555A > G mutation has been found to be responsible for both aminoglycoside-induced and nonsyndromic hearing loss in many families worldwide [4-10]. On the other hand, the 12S rRNA 1494C > T mutation has been associated with both aminoglycoside-induced and nonsyndromic hearing loss only in some Chinese and Spanish families [11-13].

The 1555A > G and 1494C > T mutations are located at the highly conserved A-site of 12S rRNA [4,11]. The A1555 and C1494 (equivalent to positions 1491 and 1409 of *Escherichia coli *16S rRNA, respectively) are in apposition to each other but do not form a base-pair. The 1555A > G or 1494C > T mutation creates a new G-C or A-U pair base-pair, thereby extending the adjacent stem by one nucleotide and making the secondary structure of mitochondrial 12S rRNA more closely resemble the corresponding region of *E. coli *16S rRNA and altering binding properties of aminoglycosides such as paromomycin, neomycin, gentamicin, and kanamycin at the A-site of 12S rRNA [14]. Thus, the administration of aminoglycosides can induce or worsen hearing loss in these subjects carrying the 1555A > G or 1494C > T mutation. In the absence of aminoglycosides, matrilineal relatives within and among families carrying the 1555A > G or 1494C > T mutation exhibited a considerable phenotypic variation with respect to severity and age-of-onset and penetrance of hearing loss [4-13]. Therefore, additional modifier factors such as aminoglycosides, nuclear and mitochondrial genetic modifiers contributed to the phenotypic variability of these mtDNA mutations [11,15-18].

However, the incidences of the 1555A > G and 1494C > T mutations were only reported in the some cohorts of hearing-impaired subjects [3,19-24]. As these mutations are only responsible for a portion of patients with hearing loss, it is anticipated that additional mutations causing hearing loss can be found in the same gene. In the present investigation, we carried out a systematic and extended mutational screening of 12S rRNA gene in a cohort of 440 hearing-impaired Han Chinese pediatric subjects from two otology clinics at Ningbo and Wenzhou, Zhejiang Province, China. Mutational analysis of 12S rRNA gene in these subjects identified the known 1555A > G and 1494C > T mutations as well as 39 other variants. Those variants have been further evaluated by phylogenetic analysis, structure-function relation and allelic frequency of these variants in the 449 Han Chinese controls from the same region. To examine if the *GJB2 *gene contributed to a deafness phenotype, we performed the mutational screening of *GJB2 *gene in 39 subjects carrying the known deafness-associated 12S rRNA mutations and 5 subjects carrying one of 5 putative 12S rRNA mutations.

## Methods

### Subjects and audiological examinations

A total of 440 unrelated hearing-impaired Chinese subjects, who were younger than 18 years old two otology clinics from Zhejiang Province, were enrolled in this study under an institutional review board-approved protocol of informed consent at the Cincinnati Children's Hospital Medical Center Institutional Review Board and Ethics Committee of Wenzhou Medical College, China. A comprehensive history and physical examination for these participating subjects were performed to identify any syndromic findings, the history of the use of aminoglycosides, genetic factors related to the hearing impairment. An age-appropriate audiological examination was performed and this examination included pure-tone audiometry (PTA) and/or auditory brainstem response (ABR), immittance testing and Distortion product otoacoustic emissions (DPOAE). The PTA was calculated from the average of the audiometric thresholds at 500, 1000, 2000, 4000 and 8000 Hz. The severity of hearing impairment was classified into five grades: normal <26 Decibel (dB); mild = 26-40 dB; moderate = 41-70 dB; severe = 71-90 dB; and profound >90 dB. The 449 control DNA used for screening for the presence of mtDNA variants were obtained from a panel of unaffected Han Chinese subjects from the same region.

### Mutational analysis of mitochondrial 12S rRNA gene

Genomic DNA was isolated from whole blood of participants using Puregene DNA Isolation Kits (Gentra Systems, Minneapolis, Minnesota, USA). Subject's DNA fragments spanning the 12S rRNA gene were amplified by PCR using oligodeoxynucleotides corresponding to positions 618-635 and 1988-2007 [25]. Each fragment was purified and subsequently analyzed by direct sequencing in an ABI 3700 automated DNA sequencer using the Big Dye Terminator Cycle (Applied Biosystems, Foster City, California, USA) sequencing reaction kit. The resultant sequence data were compared with the updated consensus Cambridge sequence (GenBank accession number: NC_012920) [26]. The homoplasmy of the 1555A > G and 1494C > T mutations in these subjects were determined as detailed previously [7,11]. The frequency of variants in the 12S rRNA gene in 449 Chinese control subjects was determined by direct sequencing of PCR products as described above.

### Mutational analysis of *GJB2 *gene

The DNA fragments spanning the entire coding region of *GJB2 *gene were amplified by PCR using the following oligodeoxynucleotides: forward-5'TATGACACTCCCCAGCACAG3' and reverse-5'GGGCAATGCTTAAACTGGC3'. PCR amplification and subsequent sequencing analysis were performed as detailed elsewhere [10]. The results were compared with the wild type *GJB2 *sequence (Version 1, GenBank accession number: M86849) to identify the mutations.

### Structural analysis

The published secondary structures for the 12S rRNA [27,28] were used to define the stem and loop structure. The secondary structure of human mitochondrial 12S rRNA was predicted by using the RnaViz program [29].

### Phylogenetic analysis

A total of 14 primate mitochondrial 12S rRNA sequences (Genbank), as shown in Table 1, were used in the interspecies analysis. These include *Homo sapiens, Gorilla gorilla, Pan paniscus, Pan troglodytes, Pongo pygmaeus, Pongo abelii, Hylobates lar, Macaca mulatta, Macaca sylvanus, Papio hamadryas, Cebus albifrons, Tarsius bancanus, Nycticebus coucang*, and *Lemur catta*. The conservation index (CI) was calculated by comparing the human nucleotide variants with other 13 primates. The CI was then defined as the percentage of species from the list of 14 different primates that have the wild-type nucleotide at that position.

**Table 1:** mtDNA sequence data of 14 primate species

Species name	GenBank accession number
*Homo sapiens*	NC_012920

*Gorilla gorilla*	NC_001645

*Pan paniscus*	NC_001644

*Pan troglodytes*	NC_001643

*Pongo pygmaeus*	NC_001646

*Pongo abelii*	NC_002083

*Hylobates lar*	NC_002082

*Macaca mulatta*	NC_005943

*Macaca sylvanus*	NC_002764

*Papio hamadryas*	NC_001992

*Cebus albifrons*	NC_002763

*Tarsius bancanus*	NC_002811

*Nycticebus coucang*	NC_002765

*Lemur catta*	NC_004025

## Results

### Study samples

The study samples consisted of 227 males and 213 females. The age of all participants ranged from 1 years old to 18 years, with the median age of 9 years. All participants were Han Chinese recruited from ENT clinics at Ningbo and Wenzhou Cities of Zhejiang Province, China. Based on a clinician review of the medical record, 98 subjects (58 males and 40 females) had a history of exposure to aminoglycosides including gentamicin, streptomycin and kanamycin, accounting for 22.3% cases of hearing loss in this cohort. These subjects, due to infections or other illness, received a conventional daily dosage of aminoglycosides (3 5 mg/kg/dose every 8 h for gentamicin or 15 25 mg/kg/dose every 12 h for streptomycin, 15 mg/kg/dose every 8 h for kanamycin) at younger than 10 years old. Hearing impairment occurred from 3 days to three months after the administration of drugs. Audiological evaluation showed that 22 subjects had severe hearing loss and 76 individuals exhibited profound hearing loss. Furthermore, there was the wide range of severity of hearing loss in 342 affected subjects who did not have a history of exposure to aminoglycosides: 149 subjects exhibited profound hearing loss, 167 subjects had severe hearing loss and 26 individuals suffered from moderate hearing loss. The onset of the hearing loss ranged from congenital to 10 years old.

### Mutational analysis of mitochondrial 12S rRNA gene

Fragments spanning 12S rRNA gene were PCR-amplified from genomic DNA of 440 hearing-impaired Chinese subjects and each fragment was purified and subsequently analyzed by DNA sequencing. Comparison of the resultant sequence with the Cambridge consensus sequence [26] identified 41 nucleotide changes in the 12S rRNA gene as shown in Table 2. All the nucleotide changes were verified by sequence analysis of both strands and appeared to be homoplasmy. Of these, 2 subjects with profound hearing loss carried the 1494C > T mutation. Both subjects carrying the 1494C > T mutation had a history of exposure to aminoglycosides. These translate to a frequency of ~0.45% for the 1494C > T mutation in this Chinese pediatric deafness population. Among these, 33 hearing-impaired subjects carrying the 1555A > G mutation were composed of 21 subjects who had a history of exposure to aminoglycosides and 12 individuals who did not receive aminoglycoside treatment. These translate to a frequency of ~7.5% for the 1555A > G mutation in this entire Chinese pediatric deafness population, and approximately 21.4% in cases of aminoglycoside ototoxicity in this Chinese pediatric population. Furthermore, 4 subjects harbored the known deafness-associated 1095T > C mutation [30,31] and 11 subjects carried the putative deafness-associated mutations at position of 961 (961insC and 961T > C) [7,21,32,33], respectively.

**Table 2:** Variants in the mitochondrial 12S rRNA gene in 440 hearing-impaired Han Chinese subjects

Position	Replacement	Conservation **index (%)**^**a**^	WC **base-pairs**^**b**^	Previously **reported**^**c**^	Number of affected subjects	Percentage (%)	Number of controls (number/449)	Percentage (%)
663	A to G	78.6	↓A-U	Yes	15	3.40	5	1.1
681	T to C	85.7	↓U-A	Yes	5	1.13	8	1.8
709	G to A	64.3	↓G-C	Yes	90	20.41	102	22.7
723	A to G	28.6		Yes	2	0.45	2	0.4
735	A to G	78.6		Yes	2	0.45	5	1.11
**747**	**A to G**	**100**	**↓A-U**	**No**	**1**	**0.23**	**0**	**0**
752	C to T	100		Yes	26	6.12	17	3.8
789	T to C	85.7		Yes	1	0.23	1	0.2
813	A to G	28.6		Yes	1	0.23	0	0
827	A to G	92.9		Yes	16	3.63	12	2.7
**839^d^**	**A to G**	**78.6**	**↓A-U**	**Yes**	**1**	**0.23**	**0**	**0**
929	A to T	42.9	↓A-U	No	1	0.23	0	0
942	A to G	64.3		Yes	1	0.23	0	0
951	G to A	92.9	↓G-C	Yes	2	0.45	2	0.4
953	T to C	57.1		Yes	**1**	0.23	0	0
**961**	**insC**	**42.9**		Yes	**9**	**2.04**	**14**	**3.1**
961	T to C	42.9		Yes	2	0.23	4	0.9
980	T to C	64.3	↓U-A	Yes	3	0.68	0	0
990	T to C	71.4	↓U-A	Yes	1	0.23	0	0
1005	T to C	35.7		Yes	21	4.76	22	4.9
1009	C to T	21.4		Yes	6	1.36	8	1.8
**1027**	**A to G**	**92.9**		**Yes**	**1**	**0.23**	**0**	**0**
1041	A to G	42.9		Yes	2	0.45	4	0.9
1048	C to T	57.1		Yes	10	2.27	11	2.4
**1095**	**T to C**	**92.9**	**↓U-A**	Yes	**4**	0.91	**1**	**0.2**
1107	T to C	85.7		Yes	36	8.39	25	5.6
1119	T to C	50.0		Yes	13	2.95	17	3.8
1187	T to C	57.1		Yes	1	0.23	0	0
1282	G to A	71.4		Yes	1	0.23	0	0
**1310**	**C to T**	**85.7**	**↓G-C**	**Yes**	**1**	**0.23**	**0**	**0**
1382	A to C	92.9	↓A-U	Yes	14	3.17	9	2.0
1391	T to C	64.3		Yes	1	0.23	1	0.2
1393	G to A	28.6	↑A-U	Yes	2	0.45	0	0
**1413**	**T to C**	**78.6**	**↑C-G**	**Yes**	**1**	**0.23**	**0**	**0**
1442	G to A	42.9		Yes	1	0.23	0	0
1462	G to A	50.0		Yes	1	0.23	**0**	**0**
**1494**	**C to T**	**78.6**	**↑U-A**	**Yes**	**2**	**0.45**	**0**	**0**
1503	G to A	50.0	↑A-U	Yes	1	0.23	0	0
1541	T to C	78.6		Yes	6	1.36	4	0.9
**1555**	**A to G**	**85.7**	**↑A-U**	**Yes**	**33**	**7.5**	**0**	**0**
1598	G to A	50		Yes	12	2.72	9	2.0

^a^The conservation index (CI) was then defined as the percentage of the human nucleotide variants with other 14 primates that have the wild-type nucleotide at that position

^b^Classic Watson-Crick (WC) base pair: created (↑) or abolished (↓).

^c^See Ruiz-Pesini E, Wallace DC (2006) and http://www.mitomap.org; http://www.genpat.uu.se/mtDB

^d^Known and putative pathogenic variants are indicated in boldface.

**Figure 1:**
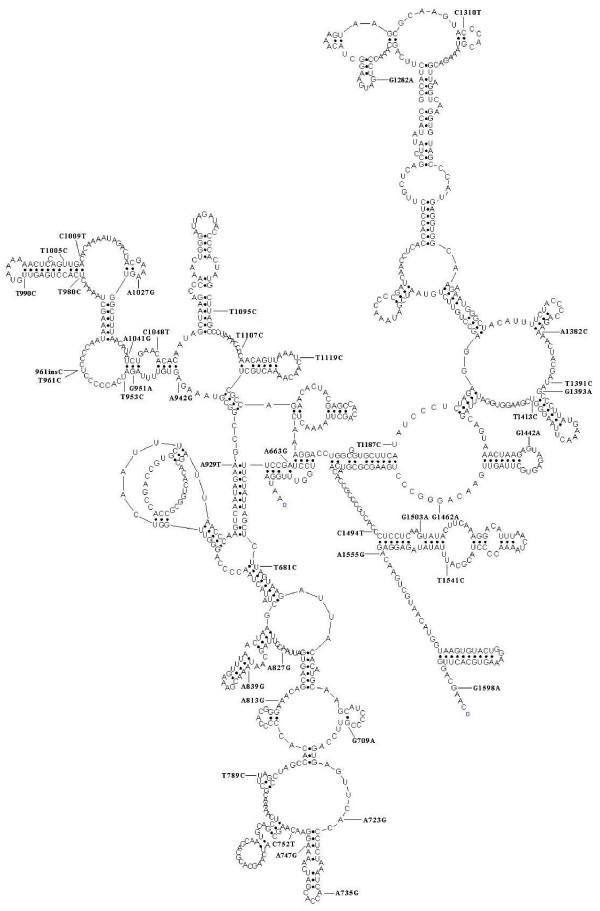
**Structure and sequence variants of human mitochondrial 12S rRNA**. The secondary structure was predicted by using the RnaViz program (De Rijk and De Wachter, 1997). The variants were indicated by arrows.

In addition to the mutations mentioned above, there were 34 known and 2 novel variants in the 12S rRNA gene [34]. These variants were first evaluated by examining the allelic frequency in 449 Han Chinese control population. Nineteen out of 41 variants were absent in this Chinese control population. Of other 22 variants, the frequencies of 8 variants were <1% in 449 Chinese controls, while the allelic frequency of other 14 variants was >1% in this control population. Furthermore, we used the secondary structure of 12S rRNA [29,35] to localize each variant with either a stem or a loop and to analyze if the base changes within stems alter classic Watson-Crick (WC) base pair [29,35]. As shown in Figure 1, 23 variants were located at the loops, while 18 variants occurred in the stems of this rRNA. As shown in Table 2 and Figure 1, 5 variants 1393G > A, 1413T > C, 1494C > T, 1503G > A and 1555A > G created a putative base-pairing(s), while 12 variants 663A > G, 681T > C, 709G > A, 747A > G, 839A > G, 929A > T, 951G > A, 980T > C, 990T > C, 1095T > C, 1310C > T and 1382A > C abolished a putative base pairing(s). This suggested that the nucleotide variants were more frequent in loops than in stems. In addition, phylogenetic analysis was performed by comparing the human 12S rRNA nucleotide variants with other 13 primates. As shown in Table 2, conservation index (CI) among the variants ranged from 21.4% (1009C > T variant) to 100% (752C > T and 747A > G variants). In particular, CI of 18 variants including 1555A > G and 1494C > T mutations were >78%, CI of other 13 variants was between 78% and 50% and CI for the remaining variants was <50%. In addition to the 1555A > G and 1494C > T mutations, the novel 747A > G variant and the known 839A > G, 1027A > G, 1310C > T and 1413T > C variants [22,34], which are absent in the 449 Chinese controls and whose CIs were >78%, were the putative deafness-associated variants. On the other hand, other variants such as 663A > G, 681T > C, 735A > G, 752C > T, 827A > G, 1107T > C and 1382A > C, whose CIs were >78%, which were present in the controls, appeared to be the polymorphisms.

### Clinical characterization of 39 hearing-impaired Chinese subjects carrying one of known or 12S rRNA mutations

Comprehensive medical evaluations of 33 probands carrying the 1555A > G mutation, two subjects harboring the 1494C > T mutation and four individuals carrying the 1095T > C mutation showed no other clinical abnormalities, including diabetes, muscular diseases, visual loss and neurological disorders. As shown in Table 3, audiological assessments of 33 subjects carrying the 1555A > G mutation showed that 15, 3 and 3 subjects with the aminoglycoside treatments exhibited profound, severe or moderate hearing loss, respectively. Moreover, 12 individuals, who did not have a history of exposure to aminoglycosides, exhibited a variety of severity and age-of-onset of hearing impairment. The age-of-onset of hearing loss in these subjects ranged from infant to 18 years, with an average of 6 years. Audiometric studies showed that 3 individuals suffered from profound hearing impairment, 5 subjects exhibited severe hearing impairment, 2 probands had moderate hearing impairment and 2 subjects exhibited mild hearing impairment. Furthermore, two subjects carrying the 1494C > T mutation exhibited severe or profound hearing loss, respectively. Among four subjects carrying the 1095T > C mutation, two subjects who was treated with aminoglycosides had profound and severe hearing loss, respectively, while two individuals who did not have a history to exposure exhibited profound and mild hearing impairment.

**Table 3:** Summary of clinical and molecular data for 44 Han Chinese subjects carrying the putative 12S rRNA mutations.

12S rRNA mutation	*GJB2 *gene mutation	Subjects	Gender	Audiometic configuration	Age-at-onset (years)	**PTA **^**a**^**(dB)** right ear	PTA (dB) left ear	Use of drugs	Level of hearing impairment
1555A > G	polymorphism	FE003-IV-1	M	Slope	1	98	98	Yes	Profound
1555A > G	polymorphism	FE007-IV-6	M	Slope	2	100	100	Yes	Profound
1555A > G	polymorphism	FE008-III-7	F	Slope	10	58	78	No	Severe
1555A > G	polymorphism	FE0128-IV-1	F	Slope	2	102	98	Yes	Profound
1555A > G	polymorphism	FE019-IV-1	F	Slope	2	67	82	Yes	Severe
1555A > G	polymorphism	FE020-III-15	M	Slope	8	81	74	Yes	Severe
1555A > G	polymorphism	FE036-III-1	M	Slope	10	58	49	No	Moderate
1555A > G	polymorphism	FE081-III-1	M	Slope	16	50	56	Yes	Moderate
1555A > G	polymorphism	FE122-III-2	F	Slope	2	71	53	Yes	Moderate
1555A > G	polymorphism	FE141-III-1	F	Slope	2	24	30	No	Mild
1555A > G	polymorphism	FE154-III-1	F	Slope	18	61	60	No	Moderate
1555A > G	polymorphism	FE160-III-1	F	Slope	5	61	74	No	Severe
1555A > G	polymorphism	FE163-III-3	M	Flat	2	110	99	Yes	Profound
1555A > G	polymorphism	FE300-II-12	F	Slope	3	110	105	Yes	Profound
1555A > G	polymorphism	FE304-II-2	F	Slope	3	100	80	Yes	Profound
1555A > G	polymorphism	FE317-III-10	M	Slope	4	94	93	Yes	Profound
1555A > G	polymorphism	FE350-III-1	F	Flat	1	100	100	Yes	Profound
1555A > G	polymorphism	NB038-III-1	M	Flat	1	90	87	Yes	Profound
1555A > G	polymorphism	NB048-III-2	F	Slope	1	78	81	No	Severe
1555A > G	polymorphism	NB052-III-2	F	Flat	1	120	102	No	Profound
1555A > G	polymorphism	NB076-III-1	M	Flat	1	118	118	Yes	Profound
1555A > G	polymorphism	NB078-III-2	F	Flat	6	110	117	Yes	Profound
1555A > G	polymorphism	NB079-III-1	M	Flat	1	102	102	Yes	Profound
1555A > G	polymorphism	NB094-III-2	F	Flat	1	117	117	Yes	Profound
1555A > G	polymorphism	NB111-III-2	F	Slope	3	83	86	Yes	Severe
1555A > G	polymorphism	NB126-III-2	F	Slope	2	84	92	No	Profound
1555A > G	polymorphism	NB137-III-1	F	Slope	2	111	115	No	Profound
1555A > G	polymorphism	ZX019-II-2	F	Slope	5	59	62	Yes	Moderate
1555A > G	polymorphism	ZX022-III-3	M	Flat	2	101	102	Yes	Profound
1555A > G	polymorphism	ZX025-III-14	M	Flat	1	113	108	Yes	Profound
1555A > G	polymorphism	ZX028-IV-1	F	Slope	3	87	87	No	Severe
1555A > G	polymorphism	ZX037-II-7	M	Flat	5	30	27	No	Mild
1555A > G	polymorphism	ZX047-III-1	M	Slope	6	78	79	No	Severe
1494C > T	polymorphism	FE247-III-1	M	Flat	3	100	100	Yes	Profound
1494C > T	polymorphism	NB133-II-1	M	Slope	2	86	88	Yes	Severe
1095T > C	polymorphism	FE312	F	Slope	9	82	80	Yes	Severe
1095T > C	polymorphism	NB021	M	Slope	10	36	37	No	Mild
1095T > C	polymorphism	NB067	M	Flat	1	93	93	No	Profound
1095T > C	polymorphism	NB100	F	Flat	5	100	95	Yes	Profound
747A > G	polymorphism	NS016-III-4	M	Flat	1	100	80	Yes	Profound
839A > G	polymorphism	NB005-III-1	F	Flat	1	78	81	Yes	Severe
1027A > G	polymorphism	FE239-II-1	M	Slope	18	82	85	No	Severe
1310C > T	polymorphism	NS071-IV-1	M	Flat	1	91	92	No	Profound
1413T > C	235DelC/299DelAT	ZX039-IV-1	F	Flat	1	114	111	No	Profound

^a ^PTA: pure-tone audiometry; dB: decibel.

### Clinical and genetic characterization of 5 hearing-impaired Chinese subjects carrying one of 5 putative 12S rRNA mutation

**Figure 2:**
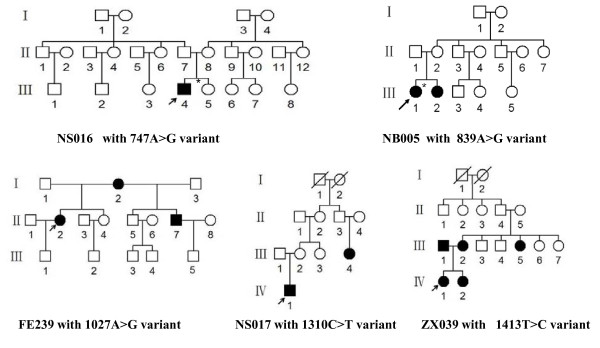
**Five Han Chinese pedigrees with aminoglycoside-induced and nonsyndromic hearing impairment**. Hearing impaired individuals are indicated by filled symbols. Arrowhead denotes probands. Asterisks denote individuals who had a history of exposure to aminoglycosides.

Comprehensive medical histories of 5 probands carrying one of 5 putative 12S rRNA mutations and other members in these families showed no other clinical abnormalities, including diabetes, muscular diseases, visual loss and neurological disorders. As shown in Table 3, two subjects received a regular dose of gentamicin for various illnesses at the age of 1 year, while other three subjects did not have a history of exposure to aminoglycosides. There was no evidence that these subjects had any known cause to account for hearing loss. Audiological examination indicated that 2 subjects suffered from severe hearing loss and 3 subjects exhibited profound hearing loss. Variable patterns of audiometric configurations were detected in these subjects: 1 subject with slope-shaped pattern and 4 individuals with flat-shaped pattern. Besides the proband, no one of the NS016 pedigree carrying the 747A > G variant suffered from hearing loss. The pedigree FE239 with three matrilineal affected relatives carrying the 1027A > G mutation showed suggestively maternally transited hearing loss. Furthermore, two matrilineal relatives of 14 members in the pedigree NB005 carrying the 839A > G mutation, as shown in Figure 2, suffered from hearing loss. In addition, four of 16 members in the pedigree ZX039 carrying the 1413T > C variant experienced the loss of hearing.

### Mutational analysis of *GJB2 *gene

To examine if the *GJB2 *gene contributed to a deafness phenotype, we performed the mutational screening of *GJB2 *gene in 39 subjects carrying the known deafness-associated 12S rRNA mutations and 5 subjects carrying one of 5 putative 12S rRNA mutations. As shown in Table 3, the subject ZX039-IV-1 carrying the 12S rRNA 1413T > C mutation harbored the known 235DelC/299DelAT mutation in the *GJB2 *gene [36,37], while none of other mutations in *GJB2 *gene was detected in other 43 affected subjects. Indeed, the absence of mutation in the *GJB2 *gene in those subjects with hearing impairment indicated that the *GJB2 *gene did not contribute to the deafness phenotype in those subjects.

## Discussion

The cohort of Chinese pediatric hearing-impaired subjects consisted of 98 subjects with aminoglycoside ototoxicity and 342 subjects, who did not have a history of exposure to aminoglycosides. Of known deafness-associated 12S rRNA mutations, the 1555A > G mutation accounted for 7.5% cases of this Chinese clinical population, while incidences of this mutation were 1.76% and 3.96% in two large cohorts of hearing impaired pediatric Han Chinese subjects from schools of deaf children [22,36]. In the present study, the incidences of the 1555A > G mutation were 2.7% and 21.4% cases of nonsyndromic and aminoglycoside-induced hearing loss, respectively. In fact, the incidences of the 1555A > G mutation varied among different ethnic origins. With regard to the subjects with aminoglycoside ototoxicity, the incidences of the 1555A > G mutation were 33% in a small Japanese cohort [19] 13%, 10.4% and 5% in three Chinese cohorts [3,21,22] and ~17% in the two white cohorts from United States and Spain [5,32,33]. However, the incidence of 1555A > G mutation in nonsyndromic hearing loss was much lower than in those with aminoglycoside ototoxicity. In two white cohorts with nonsyndromic hearing loss, the frequency of the 1555A > G mutation varied from 0.6% to 2.5% [20,24], while the incidence of the 1555A > G mutation in several Asian cohorts ranged from 2.9% to 5.3% [19,21-23]. Thus, the large proportion of subjects with aminoglycoside ototoxicity in this cohort may contribute to higher incidence of the 1555A > G mutation than other cohorts. On the other hand, the incidences of the 1494C > T mutation appeared to be lower than those of the 1555A > G mutation. In this cohort, two subjects carrying the 1494C > T mutation had a history of exposure to aminoglycosides. This data appeared to be higher than the previous reports that three familial cases of 1340 sporadic Spanish hearing-impaired subjects carried the 1494C > T mutation [12] and three cases of 1642 pediatric deaf children [22]. Therefore, these two known 12S rRNA mutations account for from 4% to 8% cases among these Chinese hearing-impaired populations [10].

Of other known deafness-mutations, the frequency of the 1095T > C mutation was 0.91% in this cohort. The 1095T > C mutation, whose CI was 92.9%, occurred in one of 449 Chinese controls. This mutation has been found in several genetically-unrelated families with nonsyndromic and aminoglycoside-induced hearing loss [21,22,30,31]. This T-to-C transition disrupted an evolutionarily conserved base-pair at stem loop of the helix 25 of 12S rRNA [27]. This nucleotide is also located at the P-site of ribosome, suggesting an important role in the initiation of mitochondrial protein synthesis [31]. Furthermore, the frequency of mutations at position 961 including 961insC and 961T > C was 2.27% in this pediatric population. Although mutations at this position have been implicated to be associated with hearing loss in different ethnic groups [21,22,32,33], the lower CI (42.9%) and presence of 4% in the controls indicated that mutations in this position were polymorphisms.

A total of 41 (39 known and 2 novel) variants in 12S rRNA gene were identified in this cohort. Similar to other mtDNA variations, these variants can be grouped into three categories: neutral, adaptive and deleterious [35]. To identify putative deleterious mutation, these variants were further evaluated using following three criteria: 1). Absent in the 449 Chinese controls; 2). CI is >78%, proposed by Ruiz-Pesini and Wallace [35]; 3). Potential structural and functional alterations [22]. Among these variants, 19 variant were absent in the 449 Han Chinese controls, while the frequency of other variants ranged from 0.2% (13 variants such as 789T > C) to 22.7% (709G > A variant) in this Chinese control population. In particular, some of these variants occurring at high frequencies of both control and patient populations were the mitochondrial haplogroup specific variants [36]. These included the 663A > G variant of haplogroup A, the 827A > G and 1119T > C variants of haplogroup B4, the 709G > A and 1598G > A variants of haplogroup B5, the 1382A > C variant of haplogroup D4, the 681T > C, 752C > T, 1048C > T and 1107T > C variants of D5 haplogroup, the haplogroup F2 specific variant 1005T > C, the 1041A > G variant of haplogroup M9a, and the 1541T > C variant of haplogroup R5b [38]. Apparently, these haplogroup specific variants were adaptive or neutral but unlikely deleterious.

Phylogenetic analysis showed that CIs of 28 variants were more than 78%. Despite their higher CI, the 14 variants such as 663A > G, 681T > C, 752C > T, 735A > G, 827A > G, 1107T > C, 1382A > C and 1438A > G were present in the controls. On the other hand, the CIs for other 7 variants including 1555A > G and 1494C > T were at least 78% but these variants were absent in 449 Chinese controls. Based on the predicted secondary structure of mitochondrial 12S rRNA [27,35], 23 variants were located at the loops and 18 variants occurred in the stems of this rRNA. Among these variants, 11 variants including the 1095T > C disrupted a WC base pairing(s) of 12S rRNA, while 5 variants including the 1555A > G and 1494C > T created a novel WC base-pairing(s) of this rRNA [28,29]. In fact, the 1555A > G or 1494C > T mutation made the mitochondrial ribosome more bacteria-like [4,11,14]. Functional characterization demonstrated that the 1555A > G or 1494C > T mutation conferred sensitivity to aminoglycosides [11,15,16,18]. Thus, individuals carrying either of mutations are predisposed to hearing loss. Indeed, the novel 747A > G variant and the known 839A > G, 1310C > T and 1413T > C variants [22,34], which resided at the stems of 12S rRNA, were fitted with three criteria for the pathogenic mutations as described above. Furthermore, the 1027A > G variant, whose location was at a loop in the 12S rRNA and whose CI was 92.9%, was absent in 449 Han Chinese controls. Thus, alterations of the tertiary or quaternary structure of 12S rRNA by these putative variants may lead to significant effects on function, thereby contributing to the deafness phenotype. Genetic and clinical evaluations of these five hearing-impaired Chinese subjects carrying one of 5 putative 12S rRNA mutation were performed. The pedigree FE239 carrying the 1027A > G mutation exhibited suggestively maternally transited hearing loss, while other four pedigrees did not have a typically maternal inheritance of hearing loss. The presence of the known 235DelC/299DelAT mutation in the *GJB2 *gene in the subject ZX039-IV-1 carrying the 1413T > C mutation indicated its role in the deafness phenotype. The absence of mutation(s) in the *GJB2 *gene in other four subjects suggested the involvement of other modifier factors in the phenotypic manifestation of these putative deafness-associated 12S rRNA variants, as in the case of these families carrying the 1555A > G mutation [39]. Further genetic and biochemical characterizations were necessary for the understanding the pathophysiology of these putative deafness-associated 12S rRNA mutations. Moreover, approximately 70% of subjects with aminoglycoside-indece hearing loss in this cohort did not carry the pathogenic 12S rRNA 1555A > G and 1494C > T mutations as well as putative deafness-associated 12S rRNA mutations. These data implicated the involvement of other nuclear genes, besides mitochondrial 12S rRNA mutations, in development of hearing loss in these subjects.

## Conclusions

Mutations in mitochondrial 12S rRNA gene accounted for approximately 30% cases of aminoglycoside-induced hearing loss in this cohort. These results strongly support the idea that the mitochondrial 12S rRNA is the hot spot for mutations associated with aminoglycoside ototoxicity. These data have been providing valuable information and technology to predict which individuals are at risk for ototoxicity, to improve the safety of aminoglycoside antibiotic therapy, and eventually to decrease the incidence of deafness.

## Competing interests

The authors declare that they have no competing interests.

## Authors' contributions

The work presented here was carried out in collaboration between all authors. ZS, BC, GP, YZ, CZ, JZ, TC LJ participated in the clinical data collection. JZ, TZ, SG, RL, LY performed the mitochondrial 12S rRNA sequence analysis and data collection. JL participated in the design of the study. MXG conceived of the study, participated in its design and coordination and drafted the manuscript. All authors read and approved the final manuscript.
